# Development of a risk assessment tool for Japanese sex offenders: The Japanese Static‐99

**DOI:** 10.1002/npr2.12330

**Published:** 2023-03-13

**Authors:** Takayuki Harada, Kazutaka Nomura, Hironori Shimada, Norito Kawakami

**Affiliations:** ^1^ Faculty of Human Sciences University of Tsukuba Tokyo Japan; ^2^ University of Tokyo Tokyo Japan; ^3^ School of Allied Health Sciences, Kitasato University Kanagawa Japan; ^4^ Faculty of Human Sciences Waseda University Saitama Japan; ^5^ Department or Digital Mental Health Graduate School of Medicine, The University of Tokyo Tokyo Japan; ^6^ Junpukai Foundation Okayama Japan

**Keywords:** paraphilic disorders, reliability and validity, risk assessment, risk factors, sex offenders

## Abstract

In Japan, sexual offending, especially paraphilic sexual offending, has become a major problem, and approximately 3000 people are arrested for frotteuristic and voyeuristic behavior each year. Considering the repetitive nature of such behaviors, determining the recidivism risk is imperative. Globally, Static‐99 is one of the most widely used actuarial risk assessment tools to predict recidivism among sex offenders. However, sexual offending is largely influenced by social and cultural backgrounds, and whether risk factors identified in the West are applicable to other countries is unknown. Therefore, we developed a Japanese version of the Static‐99 and examined its reliability and validity with 167 Japanese paraphilic sex offenders. The results showed good internal consistency (Cronbach's alpha coefficient = 0.88) and predictive accuracy (area under the curve = 0.76). The results indicate that the Japanese Static‐99 can be used with Japanese sex offenders. Moreover, risk factors identified in the Western context are applicable to Japanese sex offenders despite the different nature and manifestations of their offending.

## Abstract 2

In Japan, paraphilic sex offence is a major problem. We developed a Japanese Static‐99, a sex offender risk assessment tool to screen high risk sex offenders and to provide them with appropriate treatment. The results showed the Japanese Static‐99 has good predictive accuracy.

## Graphical abstract

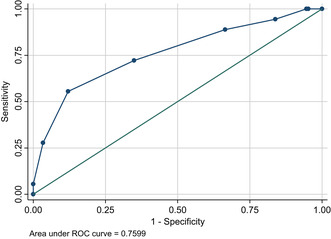

## 1 INTRODUCTION

Sexual offending is a major social problem in many countries. In Japan, paraphilic sex crimes have become a serious problem. National statistics indicate that, annually, approximately 3000 males are arrested for paraphilic sexual offenses. These individuals are more likely to reoffend than non‐paraphilic sex offenders, with approximately 30% reoffending within 5 years.^1^ Paraphilic disorders are characterized by recurrent and intense sexual arousal and manifest as deviant sexual fantasies, urges, and behaviors; these encompass frotteuristic, voyeuristic, and pedophilic disorders,^2^ and many forms of these paraphilic behaviors are criminal acts.^3^, ^4^

Only a few studies have examined the risk factors for sexual offending, especially in Asia. Sexual behaviors, including sexual offenses, are widely influenced by cultural and social factors. As mentioned above, sexual offenses in Japan have unique characteristics and are quite different in their manifestations from those observed in many Western countries. The majority of sexual offenses in Japan are relatively less violent and more paraphilic, such as frotteuristic behavior in a crowded commuting train and taking indecent photos at crowded train stations.^5^

Several aspects of the social and cultural background are significant with regard to the unique sexual offending in Japan. First, more than 10 million people commute daily using public transportation in major metropolitan areas, including Tokyo, Osaka, and Nagoya. Rush‐hour trains in the morning and evening are extremely crowded. Second, victims are less likely to report their victimization. The victim survey conducted by the National Police Agency found that only 2.6% reported the crime.^6^ Further, the survey revealed that the vast majority of victims are women, who are scared and embarrassed when they are sexually molested by strangers in public places and, therefore, do not report the incidents.

These paraphilic offenses are likely to be repeated because offenders lose control over their sexual behaviors. Many researchers and practitioners are of the view that these repetitive sexual behaviors can be considered “sexual addiction.”^5^, ^7^, ^8^, ^9^ Currently, no established diagnostic criteria exist for sexual addiction. However, it has been proposed that major clinical characteristics include (1) persistent intensive sexual preoccupation, arousal, fantasies, urges, and behaviors; (2) considerable time spent preparing for the sexual behavior, engaging in the behavior, and recovering from sexual experiences; (3) clinically significant distress or impairment arising from these behaviors; (4) persistence of these behaviors despite negative consequences; and (5) a lack of control over sexual urges and behaviors.^10^

Considering the persistent and repetitive nature of these conditions and the fact that individuals lose control over their sexual urges and behaviors, it is evident that treatment is necessary to prevent recidivism. If such sexual behavior constitutes a crime, then punishment is certainly essential. However, many studies have shown that punishment alone does not prevent recidivism and treatment has been proven to be effective in preventing it.^11^, ^12^, ^13^, ^14^, ^15^, ^16^ For example, Bonta and Andrews^11^ maintain that for effective punishment, it must be delivered immediately after the offending behavior, with appropriate intensity and certainty, while at the same time allowing the offender to learn alternative behaviors to the inappropriate behavior, all of which is almost impossible in the criminal justice system. In contrast, several meta‐analyses have found that cognitive‐behavioral therapy and pharmacotherapy, including anti‐androgen agents and anti‐depressants, are effective.^13^, ^17^, ^18^, ^19^, ^20^, ^21^, ^22^, ^23^

Risk assessment is essential for successful treatment and informed clinical decisions for sex offenders.^16^, ^24^, ^25^, ^26^ Traditionally, risk assessment relies solely on subjective clinical judgment, with predictive accuracy not exceeding a chance level.^11^ In contrast, actuarial assessment has usually more precise predictive accuracy.^11^

Static‐99 is one of the most widely used actuarial risk assessment tools for sex offenders, with established clinically useful predictive accuracy.^27^ It has 10 “static” items, which are unchangeable demographic and historical items, including the age of offending, criminal records, and the characteristics of victims.^27^

Static‐99 has been translated into multiple languages, including French, German, Italian, and Chinese.^28^, ^29^ It is widely used, but generally in Western countries; therefore, relevant research has also been confined to these countries. However, sexual behavior and sexual offending are largely influenced by social and cultural factors. Therefore, whether the risk factors used in Static‐99 could be applied outside the West is unclear.

In the current study, we aimed to develop the Japanese version of the Static‐99 and test its reliability and validity with Japanese sex offenders. This will be significant to determine whether risk factors observed in the West could be applicable to other cultures, as well as to widen our knowledge of risk factors and the prediction of recidivism pertaining to culturally specific sexual offending.

## 2 METHODS

### 2.1 Participants

The participants were 167 treatment‐seeking male sex offenders. They sought regular treatment at a private psychiatric clinic in the Tokyo metropolitan area and were diagnosed with paraphilic disorders and/or sexual preference disorders by psychiatrists, using the Diagnostic and Statistical Manual of Mental Disorders, Fourth Edition Text Revision.^2^

### 2.2 Measures and procedure

After permission was obtained from the author of the original Static‐99, it was translated into Japanese by the first author of the current study. The second author performed a back translation into English and the third author compared it with the original, listing all discrepancies. All authors worked together to amend the translation through discussion until the final Japanese version was completed.

The Japanese version of the Static‐99 also has 10 items assessing risk factors for sexual recidivism. The major risk factors are “Being young (younger than 25 years of age),” “Never lived with intimate partners,” “Prior non‐sexual violent offenses,” “Prior sexual offenses (arrested, convicted, sentenced),” and “Non‐related victims.” The total scores can be used to place patients in one of the four risk categories: low (0–1), moderate–low (2, 3), moderate–high (4, 5), and high (above 6).^29^

Using the medical records of the participants, three research assistants scored the items retrospectively. Coding was performed according to the coding rules.^29^ Only patients' ID numbers were written on the coding sheet; other personal information was kept anonymous.

Participants were followed up for 1 year after their first clinic visit, and recidivism information during the period was obtained by self‐report, information from family members or any incidence of arrest.

All participants were provided with the same treatment using the same program based on cognitive‐behavioral therapy, with a focus on relapse prevention in the same clinic by the same therapists during the follow‐up period. However, pharmacotherapy differed depending on the presence or absence of comorbidities and other factors.

### 2.3 Statistical analysis

First, Cronbach's alpha coefficient was calculated to examine reliability by determining internal consistency. Second, the demographic variables of participants and recidivism rates were compared across the risk categories. One‐way analysis of variance was used for continuous variables, and the chi‐square test and Fisher's exact test were performed for categorical variables. Third, predictive accuracy was calculated by receiver operating characteristic (ROC) analysis, with recidivism as an outcome and the Static‐99 as an explanatory variable, and the area under the curve (AUC) was calculated.^30^ The cutoff score of the Static‐99 was also calculated by the Liu method to optimally estimate recidivism. These statistical analyses were performed using Stata version 11.1 (StataCorp.).

### 2.4 Ethics

The study procedures were carried out in accordance with the Declaration of Helsinki. The University Review Committee of Mejiro University approved the study (Approval No. HEISEI 29–025). All participants provided prior written and oral informed consent.

## 3 RESULTS

Of the 167 participants, eight (4.8%) were categorized as “low risk,” 44 (26.3%) as “moderate–low risk,” 87 (52.1%) as “moderate–high risk,” and 28 (16.8%) as “high risk.”

The demographics across the risk levels were compared (Table 1). The mean age was 36.7 ± 9.7 years for the total sample: 38.8 ± 5.9 years in the low‐risk group, 37.3 ± 9.3 years in the moderate–low‐risk group, 37.6 ± 9.0 years in the moderate–high‐risk group, and 33.8 ± 11.9 years in the high‐risk group. No significant difference existed across the groups (*F* (3, 162) = 1.10, *p* = 0.35). With regard to education level, except in the high‐risk group (28.6%), more than half of the participants in the other groups had at least a college education (low: 75.0%; moderate–low: 56.8%; moderate–high: 54.0%), but no statistical difference was found across the four risk categories (*χ* ^2^ (9) = 12.67, *p* = 0.18).

**TABLE 1:** Risk level and participant demographics.

	Risk level				Statistics
	Low scores 0–1 (*n* = 8)	Low–mod scores 2–3 (*n* = 44)	Mod–high scores 4–5 (*n* = 87)	High scores ≥6 (*n* = 28)	
Age in years (SD)	38.8 (5.9)	37.3 (9.3)	37.6 (9.0)	33.8 (11.9)	*F* (3, 162) = 1.10
Education (%)					
≤ 9	0 (0.0)	1 (2.3)	2 (2.3)	3 (10.7)	*χ* ^2^ (12) = 12.67
10–14	2 (25.0)	16 (36.4)	28 (32.2)	15 (53.6)	
≥ 15	6 (75.0)	25 (56.8)	47 (54.0)	8 (28.6)	
Unknown	0 (0.0)	2 (4.5)	10 (11.5)	2 (7.1)	
Marital status (%)					
Single	2 (25.0)	12 (27.3)	40 (46.0)	28 (100.0)	*χ* ^2^ (12) = 46.61**
Married	6 (75.0)	23 (52.3)	37 (42.5)	0 (0.0)	
Divorced	0 (0.0)	7 (15.9)	10 (11.5)	0 (0.0)	
Unknown	0 (0.0)	2 (4.5)	0 (0.0)	0 (0.0)	
Employment					
Employed	6 (75.0)	29 (65.9)	51 (58.6)	7 (25.0)	*χ* ^2^ (12) = 21.24*
Student	0 (0.0)	3 (6.8)	10 (11.5)	8 (28.6)	
Not employed	2 (25.0)	12 (27.3)	22 (25.3)	13 (46.4)	
Unknown	0 (0.0)	0 (0.0)	4 (4.6)	0 (0.0)	
Problem behavior (%)					
Frotteurism	0 (0.0)	19 (43.2)	57 (65.5)	13 (46.4)	*χ* ^2^ (12) = 82.85**
Voyeurism	2 (25.0)	18 (40.0)	22 (27.3)	12 (42.9)	
Exhibitionism	0 (0.0)	2 (4.5)	5 (3.0)	2 (7.1)	
Pedophilia	0 (0.0)	4 (9.1)	0 (12.1)	0 (0.0)	
Others	6 (62.5)	1 (0.0)	3 (9.1)	1 (0.0)	
Co‐morbidity (%)					
Depression	1 (12.5)	2 (4.5)	10 (11.5)	0 (0.9)	*χ* ^2^ (12) = 27.30**
Developmental disorders	1 (12.5)	0 (0.0)	15 (17.2)	11 (39.3)	
Intellectual disorders	0 (0.0)	0 (0.0)	2 (2.3)	4 (14.3)	
Other addiction	0 (0.0)	0 (0.0)	5 (5.7)	1 (3.6)	
Others	0 (0.0)	0 (0.0)	2 (2.3)	1 (3.6)	
Relapse (%)^a^	0 (0.0)	2 (4.5)	6 (6.9)	10 (35.7)	*χ* ^2^ (3) = 14.43**

Abbreviation: SD, standard deviation.

^a^ Relapse was measured for 1 year.

** *p* < 0.01.

* *p* < 0.05.

With regard to marital status (*χ* ^2^ (9) = 46.61, *p* < 0.01), employment status (*χ* ^2^ (9) = 21.24, *p* < 0.05), and distribution of problem behaviors (*χ* ^2^ (15) = 124.44, *p* < 0.01), significant differences were found. The residual analysis revealed that the proportion of married participants was significantly large in the low‐ and moderate–low‐risk categories. In the high‐risk category, all participants were single. With regard to employment, the proportion of employed individuals was significantly small and that of students was significantly large in the high‐risk group. In terms of problem behavior, frotteurism in the low‐risk group was significantly low, whereas it was significantly high in the moderate–high‐risk group. Pedophilia occurred significantly more frequently in the low–moderate‐risk group.

It is often pointed out that individuals with addictive disorders frequently have comorbid conditions. Among our participants, 55 (32.9%) had at least some comorbid disorder. These included 13 with depression, 27 with developmental disorders, 6 with mild intellectual disorders, 6 with other addictive disorders, and 3 with other disorders. There were significant differences by risk level (*χ* ^2^ (12) = 27.30, *p* < 0.00), that is, participants with higher risk were likely to have psychiatric comorbidities.

In summary, higher‐risk participants were characterized as being single, unemployed, and engaged in frotteurism.

The demographics across problem behaviors were also compared (Table 2). The mean age, education level, marital status, and employment were not significantly different. However, Static‐99 scores of frotteurists, voyeurists, and exhibitionists were significantly higher than others (*F* (5, 159) = 4.66, *p* < 0.00).

**TABLE 2:** Problem behaviors and demographics of participants.

	Frotteurism (*n* = 89)	Voyeurism (*n* = 54)	Exhibitionism (*n* = 9)	Pedophilia (*n* = 4)	Others (*n* = 11)	Statistics
Age in years (SD)	37.3 (10.4)	35.8 (9.8)	35.6 (5.1)	31.8 (6.2)	38.3 (4.8)	*F* (5, 159) = 0.56
Education (%)						
<=9	4 (4.5)	1 (1.9)	1 (11.1)	0 (0.0)	0 (0.0)	*χ* ^2^ (12) = 11.69
10–14	34 (38.2)	19 (35.2)	3 (33.3)	0 (0.0)	5 (45.5)	
15=<	42 (47.2)	31 (57.4)	3 (33.3)	4 (100.0)	6 (54.5)	
Unknown	9 (10.1)	3 (5.6)	2 (22.2)	0 (0.0)	0 (0.0)	
Marital status (%)						
Single	42 (47.2)	27 (50.0)	5 (55.6)	2 (50.0)	6 (54.5)	*χ* ^2^ (12) = 8.23
Married	33 (37.1)	24 (44.4)	2 (22.2)	2 (50.0)	5 (45.5)	
Divorced	12 (13.5)	3 (5.6)	2 (22.2)	0 (0.0)	0 (0.0)	
Unknown	2 (2.2)	0 (0.0)	0 (0.0)	0 (0.0)	0 (0.0)	
Employment (%)						
Employed	47 (52.8)	34 (63.0)	4 (44.4)	4 (100.0)	4 (36.4)	*χ* ^2^ (12) = 17.73
Student	11 (12.3)	9 (16.7)	1 (11.1)	0 (0.0)	0 (0.0)	
Not employed	28 (31.5)	11 (20.4)	3 (33.3)	0 (0.0)	7 (63.6)	
Unknown	3 (3.4)	0 (0.0)	1 (11.1)	0 (0.0)	0 (0.0)	
Static‐99 (SD)	4.17 (1.25)	4.20 (1.65)	4.77 (1.30)	2.75 (0.50)	2.27 (2.45)	*F* (5, 159) = 4.66**
Risk level (%)						
Low	0 (0.0)	2 (3.7)	0 (0.0)	0 (0.0)	6 (54.5)	*χ* ^2^ (12) = 82.85**
Low‐Med	19 (21.3)	18 (33.3)	2 (22.2)	4 (100.0)	1 (9.1)	
Med‐High	57 (64.0)	22 (40.7)	5 (55.6)	0 (0.0)	3 (27.3)	
High	13 (14.6)	12 (22.2)	2 (22.2)	0 (0.0)	1 (9.1)	
Relapse^a^ (%)	13 (14.6)	4 (7.4)	0 (0.0)	0 (0.0)	1 (9.1)	*F* (4, 161) = 1.09

Abbreviation: SD, standard deviation.

^a^ Relapse was measured for 1 year.

** *p* < 0.01.

* *p* < 0.05.

Table 3 shows the number of recidivists in each risk category. The overall recidivism rate for 167 participants was 10.8% (18 of 167). Of all the recidivists, eight (44.4%) engaged in frotteurism and two (25.0%) in voyeurism. No one in the low‐risk level reoffended (0/8), but two of the 44 (4.5%) moderate–low‐risk participants, six of the 87 (6.9%) moderate–high‐risk participants, and 10 of the 28 (35.7%) high‐risk participants recidivated. The recidivism rates across the various risk categories were significantly different (*χ* ^2^ (3) = 22.21, *p* < 0.01), and the residual analysis indicated that the proportion of recidivated high‐risk participants was significantly higher than that of participants in other risk categories.

**TABLE 3:** Risk category distribution and relapse.

	Low (*n* = 16)	Low–mod (*n* = 46)	Mod–high (*n* = 84)	High (*n* = 21)	*χ* ^2^
Relapse (%)	1 (6.3)	2 (4.3)	5 (6.0)	10 (47.6)	14.43*

* *p* < 0.01.

**FIGURE 1:**
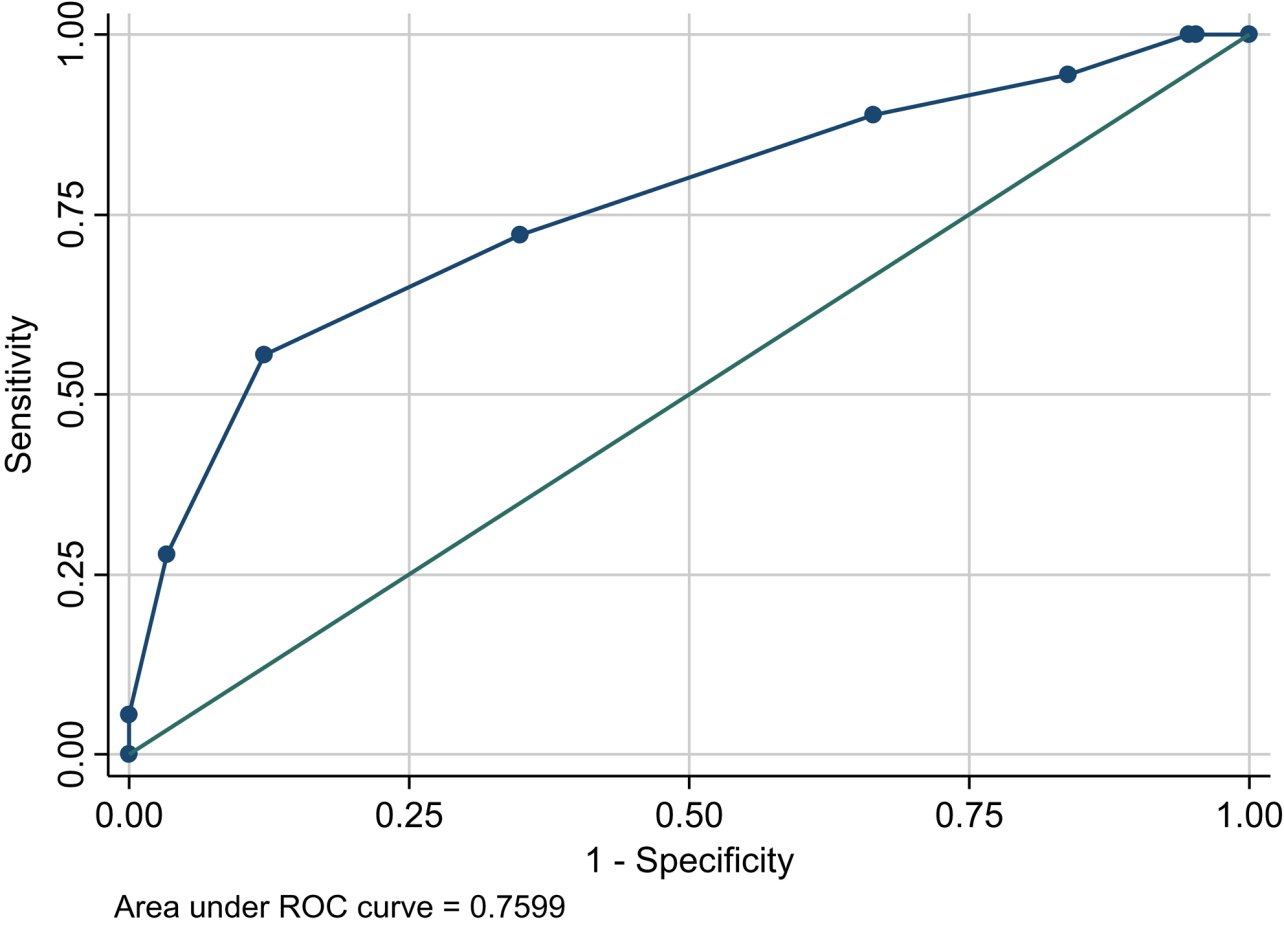
Receiver operating characteristic (ROC) curve of the Japanese Static‐99.

Finally, Cronbach's alpha coefficient was calculated and a value of 0.88 was obtained. The AUC was also calculated, and a value of 0.77 (95% CI = 0.63–0.89) was obtained; the ROC curve is shown in Figure 1. This means that there is a 77% chance that a randomly selected recidivist would have a higher score on the Static‐99 than a randomly selected non‐recidivist. Subsequently, a cutoff value of five was obtained by the Liu method. Therefore, the Japanese Static‐99 indicates that it is optimal to judge a case as having a high recidivism risk if the score is five or higher. The true positive rate was 56%, while the false positive rate was 44%. However, since this was the result of a 1 year follow‐up and treatment was provided during that period, the true positive rate could be even higher if the follow‐up period was extended or treatment was not implemented.^31^ In addition, it is important to keep the false‐negative rate as small as possible in predicting the risk of sexual offenses to minimize victimization.^11^ Since the false‐negative rate of this scale is kept as low as 12%, the risk of false negatives is considered to be considerably limited.

## 4 DISCUSSION

### 4.1 International comparison of demographics and recidivism risk

First, with regard to risk level, most participants (62.9%) were classified as low to moderate‐high risk. This is not surprising given the fact that most of them had been arrested multiple times in the past and had visited our clinic for treatment because they were unable to control their problematic sexual behavior on their own. We also found that a large percentage of the high‐risk participants were relatively young, single, and unemployed. However, being young and single as well as having a previous criminal record were included in the Static‐99 items, which means that they were inevitably judged to be at high risk.

In addition, we found that the higher risk participants are more likely to have psychiatric comorbidities. A previous study indicated that a considerable number of individuals with paraphilic disorders and sex offenders have comorbid psychiatric disorders, including mood disorders, developmental disorders, personality disorders, and substance use disorders.^32^ However, these data come from a small number of studies usually based on small sample sizes or case reports.^33^ Further research is required to clarify these associations.

We also examined how Japanese paraphilic sex offenders differ from those in previous foreign studies. It should be noted, however, that accurate comparisons with other studies are difficult because of differences in the characteristics of the study participants and the study setting.

In a study of 409 sex offenders in Canadian prisons, the mean age of recidivists was 36.3 ± 11.2 years and that of non‐recidivists was 31.1 ± 11.0 years.^34^ In a study of 194 child molesters in the US, the mean age at arrest was 31.5 ± 10.3 years for recidivists and 37.8 ± 12.4 years for non‐recidivists.^35^ In addition, 344 inpatients in a Canadian mental hospital (a maximum‐security institution) had a mean age of 36.2 ± 10.9 years at discharge, and 531 prisoners in the UK had a mean age of 34.4 ± 12.7 years at release.^27^ In comparison to the participants in this study, they all shared the same age range of early to mid‐30s. Since it is said that the onset of problematic sexual behavior is usually prior to age 18 and it peaks in the 20s to 30s,^36^ and since a considerable number of years is expected to elapse after that before arrest and incarceration by sexual offending, it is reasonable that the participants in all countries are mainly in their 30s or older.

Regarding marital status, 59.6% of Canadian recidivists and 62.8% of non‐recidivists were married. For US child molesters, 54.2% of recidivists and 29.0% of non‐recidivists were married. The overall percentage of married participants in this study was 39.5% and that of low‐risk participants was 75.0%, while that for the high‐risk group was 0%. Moreover, including the divorced, 49% of the participants had been married before. Although the low rate of married high‐risk participants is striking, being single is one of the Static‐99 items. On average, there was no significant difference between the current study and previous studies in terms of marital status.

While there have been several previous studies on the demographics and characteristics of sex offenders, almost all studies were conducted in North America and Europe. Therefore, the findings of this study revealed that there were no significant differences in the characteristics of sex offenders between Japan and Western countries, even though the manifestation of their offenses was different.

### 4.2 The reliability and predictive validity of the Japanese Static‐99

Through this study, we aimed to develop a psychometrically sound risk assessment tool for Japanese paraphilic sex offenders.

Cronbach's alpha coefficient indicated that the Japanese Static‐99 had a high level of internal consistency; it was thus shown to be reliable.

In terms of predictive validity, the Japanese Static‐99 showed moderate‐to‐high predictive accuracy, and its AUC was slightly larger than that of the original Static‐99 (0.76 vs. 0.71).^37^ Individuals assessed as “high risk” were more likely to reoffend, and approximately one‐third of them actually reoffended in 1 year. Conversely, no participants assessed as “low risk” reoffended during the study period. It can thus be concluded that the Japanese Static‐99 is as effective as the original Static‐99 in ranking the relative risk of sex offenders and predicting future reoffending and its predictive validity is supported.

In comparison to previous studies on recidivism, the original Static‐99 sought data on recidivism rates after 5 years for sex offenders in Canadian and British prisons. It is important to note that our study collected recidivism rates after 1 year and that simple comparisons are difficult to make because of the vastly different nature of the sex offending.

The original Static‐99 data showed 257 (23.7%) low‐risk individuals with a 5‐year recidivism rate of 5.6%, 410 (37.8%) moderate‐low risk individuals with a 19.6% recidivism rate, 290 (26.7%) moderate‐high risk individuals with a 28.4% recidivism rate, and 129 (11.9%) high‐risk individuals with a 39.0% recidivism rate.^31^

Compared to the present study, there was a significant difference in the proportion of each risk level (*χ* ^2^ (3) = 44.38, *p* < 0.01), with the original study having a significantly lower proportion of moderate‐high‐risk individuals.^29^ The present study had significantly more moderate‐high and high‐risk individuals and significantly fewer low‐risk and moderate‐low‐risk individuals. Furthermore, recidivism rates were higher for high‐risk offenders in the present study sample, despite the shorter observation period.

This may be related to the fact that the focus of this study was placed on paraphilic sex offenders. For example, the Static‐99 rated a higher risk when the victim was a stranger. Since frotteurism and voyeurism often involve a stranger, the risk for Japan's sex offenders tends to be rated higher. It is important to note that the Static‐99 does not determine the risk of maliciousness or seriousness of sexual offenses, but only the risk of recidivism. In the case of frotteurism and voyeurism, the relative magnitude of harm and seriousness caused by offending is smaller than that of rape and another violent offending. However, the recidivism risk is higher. In fact, crime statistics in Japan indicated that the 5‐year recidivism rate for rape was 0.7%, while that for frotteurism and voyeurism was 35.1%.^38^

Furthermore, when compared to the predictive validity in previous studies in North America and Europe, the US study produced an AUC value of 0.74^39^; the UK,0.73^40^; and Sweden, 0.76.^41^ Thus, the AUC value obtained in this study is comparable, and we can make risk predictions for sex offenders in Japan with some confidence.

However, these AUC values obtained from the Static‐99 do not indicate a very high predictive accuracy.^31^ This is because the Static‐99 does not necessarily cover all recidivism risk factors and does not include dynamic factors such as personality and cognition. However, the Static‐99 has clinical utility. For example, it can be used relatively easily by practitioners working in forensic and medical settings, even if they have no psychological background. Although understanding of coding rules is essential and training in coding is strongly recommended, the Static‐99 coding is easier compared to other psychological assessments, and in most cases does not even require an interview with the offender.^31^ Given this clinical utility and the scientific soundness of the Static‐99, it should be widely used in Japan. For cases that require close examination, other dynamic factors should be assessed by experts to further improve their predictive accuracy.

The results indicated that risk factors for sexual offending identified in Western studies^11^, ^25^, ^27^, ^28^ are also applicable to Japanese paraphilic sex offenders despite considerable differences in the nature and manifestations of their offending. It is suggested that sex offenders differ from culture to culture, but they share more similarities than differences. For example, the results show that early‐onset sex offenders without any intimate relationships are more likely to reoffend, and those with multiple offenses, especially violent ones, are most dangerous and have many treatment needs. These findings also support the construct validity of the Japanese Static‐99.

Several clinical implications can be considered using the Static‐99 scores. Treatment frequency and duration should be changed according to the risk level. In particular, for high‐risk individuals, more intensive medical services such as residential treatment, daycare support, participation in self‐help group meetings, and medication may be necessary.^11^, ^22^, ^42^, ^43^, ^44^ Moreover, the contents of treatment programs require some modification according to risk level. For example, low‐risk individuals may need only relapse prevention interventions, while high‐risk individuals may need more interventions such as anger management, stress coping, and interpersonal skills and empathy training to address multiple treatment needs.^12^, ^42^, ^45^ The Japanese Static‐99 can provide such clinical information to allocate offenders to the relevant risk levels, on the basis of which they can receive the appropriate treatment.

This study has several limitations. First, the follow‐up period may be short; recidivism studies usually follow participants for 3–5 years.^18^ However, considering the fact that more than half of those with frotteurism reoffended within 1 year after their first offense, a 1‐year follow‐up is not too short.

Second, this study was conducted retrospectively, and substantial relevant information was missing from the medical records. Although the Static‐99 does not require an additional interview with a patient, medical records should include enough information to enable proper coding.

Third, participants were recruited from patients visiting medical institutions, and a criminal justice population was excluded. A considerable number of sex offenders undergo criminal punishment and they may be reluctant to receive medical treatment.^46^ Thus, the participants of this study may be biased. Moreover, the study participants were provided with treatment during the study period, unlike the studies conducted in the prison setting. This may affect recidivism. In terms of treatment, all participants received the same psychotherapy. However, some participants received pharmacotherapy depending on their conditions (e.g., presence or absence of comorbidities) in addition to psychotherapy. This may also affect the outcome.

We would like to suggest future research directions. First, the development of comprehensive risk assessment tools is necessary to obtain a more precise prediction. Hanson and Thornton emphasized that the Static‐99 was not intended to provide a comprehensive assessment.^27^ As it includes only static factors, excluding any dynamic or changeable factors associated with sexual reoffending, it can be used to decide the treatment intensity but not to obtain information on treatment needs.^28^, ^47^, ^48^ For this purpose, several other assessment tools encompassing dynamic risk factors have been developed. For more precise risk and needs assessment, clinical tools to assess dynamic risk factors should also be developed for Japanese sex offenders.

Second, the unique characteristics of Japanese sex offenders and the cultural background should be taken into consideration to improve the predictive accuracy of Static‐99. In North America, rape, sexual assault, and pedophilia are major sexual offenses.^42^, ^43^, ^49^ However, less aggressive and more paraphilic offenses are common in Japan, as mentioned previously.^5^ The Static‐99 includes items such as “Any stranger victim” and “Any unrelated victim.” Sexual offenses such as rape and child molestation are likely to victimize acquaintances and relatives. If offenders victimize a stranger, they are deemed high risk because such an offense is relatively rare and more difficult to commit. However, paraphilic offenses such as frotteurism and voyeurism (e.g., touching women in a crowded train, taking peeping photos) usually target strangers. Such a typical victimization style among Japanese sex offenders results in higher scores on the Static‐99 and may lead to an exaggeration of their recidivism risk. Given these differences in sexual offending between Japan and the West, further studies are necessary to identify specific risk factors for Japanese paraphilic sex offenders and to improve the predictive validity of Static‐99.

## AUTHOR CONTRIBUTIONS

T.H. planned and conducted the research. T.H. translated the original scale into Japanese, K.N. back‐translated the scale, and H.S. checked discrepancies between the original and back translation. T.H. performed the statistical analysis. N.K. supervised the research project. T.H. drafted the manuscript, and all authors checked the manuscript.

## FUNDING INFORMATION

This study was funded by JSPS KAKENHI (grant number JP17K04442).

## CONFLICT OF INTEREST STATEMENT

N.K. is employed at the Department of Digital Mental Health, an endowment department supported with an unrestricted grant from 15 enterprises (https://dmh.m.u‐tokyo.ac.jp/c) and reports personal fees from SBAtWork Corp., the Occupational Health Foundation, SB AtWork Corp., RIKEN, Japan Aerospace Exploration Agency, Sekisui Chemicals, Junpukai Health Care Center, and the Osaka Chamber of Commerce and Industry, neither of which have contributed to the submitted work. The other authors declare no conflict of interest.

## ETHICS STATEMENT

Ethics approval: The study procedures were carried out in accordance with the Declaration of Helsinki. The University Review Committee of Mejiro University approved the study (Approval No. HEISEI 29‐025).

Patient consent statement: All participants provided prior written and oral informed consent.

Permission to reproduce material from other sources: Not applicable.

Registry and the Registration No. of the Study/Trial: N/A.

Animal Studies: N/A.

## Data Availability

The data that support the findings of this study are openly available in Figshare at 10.6084/m9.figshare.21525156.
